# Supplementary material for: Exploring the effect of industrial agglomeration on income inequality in China
Source: PLoS One. 2023 Jun 29;18(6):e0287910. doi: 10.1371/journal.pone.0287910 (PMC10310039; doi:10.1371/journal.pone.0287910)
Supplement: Attachment — Submitted filename: Recommendations for authors PONE .docx [file pone.0287910.s001.docx]

The study aims “Exploring the effect of industrial agglomeration on income inequality in China”. The topic is interesting, however, some considerations need to be explained and after minor explanations and corrections, the paper can be published:

1. Manuscript needs to follow the journal guideline and template.
2. Use initial capitals for each key word and separate them with a comma.
3. Try to start the abstract by interdicting why are you envisioned to do this research, the abstract is very long try to write it in a precise and comprehensive way.
4. Headings mentioned in the literature section are not appropriate, try to rewrite. E.g, “Defining and measuring industrial agglomeration” this heading can be written as “Measurement of industrial agglomeration”. Instead of “The link between industrial agglomeration and income inequality” you can write “Linkages between industrial agglomeration and income inequality”. “Other determinants of income inequality” “income inequality and its determinants “.
5. Abbreviations should be written in full form at the first place. E.g GE coefficient is not clear.
6. Instead of writing “Table 1. Data”, write “Table 1. Variable descriptions”.
7. An explanation about the method used to analyze the data in the study is missing.
