# Supplementary material for: Exploring the effect of industrial agglomeration on income inequality in China
Source: PLoS One. 2023 Jun 29;18(6):e0287910. doi: 10.1371/journal.pone.0287910 (PMC10310039; doi:10.1371/journal.pone.0287910)
Supplement: Attachment — Submitted filename: Response to Reviewers.docx [file pone.0287910.s002.docx]

February 8, 2023

*PLOS ONE*

Dear Editors and Reviewers:

Thank you for your letter and for the reviewers’ comments concerning our manuscript (*PONE-D-22-32289*). Those comments are all valuable and very helpful for revising and improving our paper, as well as the important guiding significance to our researches. We have studied comments carefully and have made correction which we hope meet with approval. Revised portion are marked up using the “Track Changes” function in the paper. The main corrections in the paper and the responses to the reviewer’s comments are as following:

**Response to Reviewer 1 Comments:**

**Point 1:** Abstract: The abstract section should contain the research background, motivation, questions, methods, conclusions and insights, etc. The elaboration of the research gap can be briefly elaborated or deleted.

**Response 1:** Based on the reviewer’s comments, we have deleted “A number of studies show that if income inequality in a country is too large, it will not be conducive to the development and growth of the country, and will result in great social and political instability” and “Industrial agglomeration has accelerated economic growth, but it may also widen regional income inequality” which are not very important when we elaborate the gap in the abstract. Thanks for your valuable comments.

**Point 2:** Introduction: The introduction section, as the beginning of the article, answers the question of "why research". It is enough to explain the background, the history and status of previous research in the relevant field and the research gap, the value and the purpose of the research, the author uses five paragraphs to elaborate, which will lead to too much content and illogicality in this section, please delete unnecessary content. In addition, the introduction section lacks a description of the research objectives. Then, please add the corresponding references.e.g.line80-line82.

**Response 2:** We gratefully appreciate for your comment. We have deleted some unnecessary content. Regarding the research objective, it can be reflected in line75-line76. Additionally, several contributions in line93-line114 also explain the goals of this paper. The corresponding references in contributions have been elaborated in the following sections. Thanks for your valuable comments.

**Point 3:** Literature review: In the literature review section, the author divided into three parts, the definition and measurement of industrial agglomeration, the relationship between industrial agglomeration and income inequality, and other factors that affect income inequality. The content is abundant, but it does not summarize and condense the literature combing, the literature is seriously piled up, and the classification of each scholar's view is not sufficiently summarized. Then, please add the corresponding references.e.g.line146.

**Response 3:** We appreciate for your valuable comments. In the literature section, the beginning of each paragraph summarizes the content and reflects the classification of scholars’ view. According to the reviewer’s suggestions, we have rewritten the content in line190-line213. The literature by Maure and Sedilbt (1999) is not important in the paper. Therefore, we have deleted this literature based on the reviewer’s comments. Thanks for your valuable comments.

**Point 4:** Data and methodology : In the variable construction section, where the author compares and contrasts measures of income inequality, please insert appropriate citations to enhance the power of persuasion. In the section on control variables, please give appropriate citations to justify and demonstrate their usefulness. Also, in the “Other determinants of income inequality section” of the literature review, the factors mentioned that affect income inequality also include “economic growth, human capital, transportation, economic structure, government intervention, and unemployment”. Do they have any influence on this research and are they all included in the consideration of the control variables. This leads me to question the scientific validity and reasonableness of the third part of the literature review in the manuscript. The format of equation (2) and equation (3) is not uniform, please revise. The p-value of the indicator used to express the significance of the variables should be italicized, please check and correct it. In addition, I suggest that the spatial correlation analysis be placed in the results and discussion section.

**Response 4:** It is really true as Reviewer suggested. We have added citations to enhance the power of persuasion in line298-line301. Appropriate citations on control variables are written in the literature’s third part “Income inequality and its determinants” and the model specification’s line470-line473. Most of factors including “economic growth, human capital, transportation, economic structure, government intervention, and unemployment and so on” are considered in the control variables. For example, economic growth elaborated in the literature section is also expressed by regional economic development level in our model which is a very important control variable. However, some factors such as transportation are not significant in our empirical study in China although maybe significant in other countries. The choice of control variables in this study is seriously checked and based on China’s situation and some important literature. We appreciate for your valuable suggestions about the place of the spatial correlation analysis. In this paper, the empirical model, the spatial Durbin model is put forward after proving that dependent variable or independent variable is correlated spatially. Thus, we made the spatial correlation analysis be placed before the model specification section. Thanks for your valuable comments.

**Point 5:** Results and discussion: First, discuss results in detail and mention the innovative outcome. Further results should be backed with appropriate literature. Results should be further elaborated in detail. Usually, relevant tests need to be performed on the data before conducting empirical analysis. Considering that this manuscript uses data from 31 provinces in China, here is a question: When the authors processed the data, were the data for Tibet, Hong Kong, Macau and Taiwan complete? If not, how were you processed? If non-equilibrium panel data are used, do the corresponding tests of the data meet the requirements? Please show the results of the tests in the manuscript to prove the scientific validity of the manuscript. Moreover, Table 3 demonstrates a low R2, which confirms the possible inappropriateness of the author's choice of control variables. Also, please fix the formatting problems in Table 3.

**Response 5:** We appreciate for your valuable comments. There are some appropriate literature to support the further results in the discussion section. The data are for Tibet but not for Hong Kong, Macau and Taiwan. Because Hong Kong, Macau and Taiwan have different systems with China mainland. Additionally, we used equilibrium panel data in our study which include 31 provinces from 2003 to 2020. Therefore, the corresponding tests of the data meet the requirements. Regarding R2, when we chose the appropriate control variables, we have run the regressions from (I) to (III) and the R2 is improved greatly from 0.2788 to 0.5868, which proves the possible appropriateness of control variables. We have fixed the formatting problems in all tables. Thanks for your valuable comments.

**Point 6:** Please differentiate with the discussion section, condense the core conclusions of this manuscript and delete some contents that belong to the discussion section.

**Response 6:** We have deleted some not important contents in the conclusions section. Thanks for your valuable comments.

**Point 7:** References: Too many references cited, please make appropriate deletions.

**Response 7:** We have deleted some not important references based on the reviewer’s comments. Thanks for your valuable comments.

**Point 8:** Avoid grammatical and typo errors and revise the manuscripts for corresponding concerns.

**Response 8:** We have checked and revised the entire paper. Thanks for your valuable comments.

**Point 9:** Corresponding author information needs to be confirmed. (title page )

**Response 9:** The title page has been included into the beginning of our manuscript. There are two corresponding authors which have been added in the title page. Thanks for your valuable comments.

**Response to Reviewer 2 Comments:**

**Point 1:** Manuscript needs to follow the journal guideline and template.

**Response 1:** Based on the journal guideline and template, we have checked and revised the entire paper. Thanks for your valuable comments.

**Point 2:** Use initial capitals for each key word and separate them with a comma.

**Response 2:** We have used initial capitals for each key word and separate them with a comma based on the reviewer’s comments. Thanks for your valuable comments.

**Point 3:** Try to start the abstract by interdicting why are you envisioned to do this research, the abstract is very long try to write it in a precise and comprehensive way.

**Response 3:** We have deleted “A number of studies show that if income inequality in a country is too large, it will not be conducive to the development and growth of the country, and will result in great social and political instability” and “Industrial agglomeration has accelerated economic growth, but it may also widen regional income inequality” to make the abstract shorter according to the reviewer’s comments. Thanks for your valuable comments.

**Point 4:** Headings mentioned in the literature section are not appropriate, try to rewrite. E.g, “Defining and measuring industrial agglomeration” this heading can be written as “Measurement of industrial agglomeration”. Instead of “The link between industrial agglomeration and income inequality” you can write “Linkages between industrial agglomeration and income inequality”. “Other determinants of income inequality” “income inequality and its determinants”.

**Response 4:** We appreciate for your valuable suggestions. Thus, we have rewritten the headings in the literature section based on your comments. Thanks for your valuable comments.

**Point 5:** Abbreviations should be written in full form at the first place. E.g GE coefficient is not clear.

**Response 5:** Considering the Reviewer’s suggestion, we have added the full form of abbreviations at the first place in line162, line170-line171, line290-line291 and line327. Thanks for your valuable comments.

**Point 6:** Instead of writing “Table 1. Data”, write “Table 1. Variable descriptions”.

**Response 6:** Based on the reviewer’s suggestion, we have rewritten the title of this table. Thanks for your valuable comments.

**Point 7:** An explanation about the method used to analyze the data in the study is missing.

**Response 7:** An explanation about the method used to analyze the data is written in the section of “Data and Methodology” and concluded in line476-line486. Thanks for your valuable comments.

**Other changes:**

1. There are two corresponding authors, Suhua Zhang and Yasmin Bani. We have revised this information in the title page. Additionally, the title page has been included into the beginning of our manuscript based on your requirements.
2. The data are publicly accessible at (doi:10.5061/dryad.z08kprrht).
3. Based on the editor’s suggestions, we have changed the Figure which contains map images to Table 1 in line318-line357.
4. We have deleted some not important references in this paper according to your comments.
5. We have checked and revised the formatting problems based on the PLOS ONE style templates.

We appreciate for editors and reviewers’ warm work earnestly, and hope that the correction will meet with approval. Once again, thank you very much for your comments and suggestions.

Sincerely,

Suhua Zhang

zhangsuhua1023@gmail.com
