# Supplementary material for: Exploring the effect of industrial agglomeration on income inequality in China
Source: PLoS One. 2023 Jun 29;18(6):e0287910. doi: 10.1371/journal.pone.0287910 (PMC10310039; doi:10.1371/journal.pone.0287910)
Supplement: Attachment — Submitted filename: Response to Reviewers.docx [file pone.0287910.s003.docx]

May 30, 2023

*PLOS ONE*

Dear Editors and Reviewers:

We would like to thank the editor for giving us a chance to revise the manuscript, and thank the reviewers’ comments concerning our manuscript (*PONE-D-22-32289R1*). Those comments are all valuable and very helpful for revising and improving our paper, as well as the important guiding significance to our researches. We have studied comments carefully and have made correction which we hope meet with your approval. Revised parts are marked up using the “Track Changes” function in the paper. The main corrections in the paper and the responses to the reviewer’s comments are as following:

**Response to Reviewer #2 Comments:**

**Point:** Authors have improved the manuscript and the current version is good to be published in Plos One. Good luck.

**Reply:** We gratefully appreciate for your comments and encouragement.

**Response to Reviewer #3 Comments:**

**Point 1:** Confusion of important concepts. For example, “Industrial agglomeration” and “Industrial cluster” are not the same concept. The author must carefully read some important literature about “industrial agglomeration”, and enhance the ability of concept discrimination. What’s more, some crucial statements lack preciseness. For example, “few have studied the impacts of industrial agglomeration on income inequality, and even fewer have studied the spatial correlation of income inequality”.

**Reply 1:** Thanks to the reminding of the reviewer. Because our expression about the correlation between industrial agglomeration and industrial cluster is not accurate. Based on your suggestion, we have revised the content from “These three names are much the same” to “*These three names are closely related concepts, but they have subtle differences*”. Additionally, we have rewritten the content on “few have studied the impacts of industrial agglomeration on income inequality, and even fewer have studied the spatial correlation of income inequality”. The current content is “*few studies have been conducted on the impacts of industrial agglomeration on income inequality and their spatial correlation*”.

**Point 2:** Literature review: the part of “Measurement of industrial agglomeration” needs to be simplified. This part focuses on the main measurement methods of industrial agglomeration (brief introduction), “why does this paper choose ‘location quotient’ to measure industrial agglomeration”, and other important content. The part of “Linkages between industrial agglomeration and income inequality”: It is suggested that the logical relationship and mechanism between them should be effectively analyzed, rather than the stacking statement of the existing literature. The part of “Income inequality and its determinants”: why write this part? Some variables are not effectively reflected in the empirical analysis of this paper. So, it is not recommended to state them as a separate part. What’s more, the Literature review should focus on "income inequality ", "industrial agglomeration" and "the relationship between them".

**Reply 2:** Thanks for your valuable comments. The part of “Measurement of industrial agglomeration” concentrates on explaining why this paper choose ‘location quotient’ and introducing this method. Thus, we have added three literatures on location quotient and adjusted the expression in this part. The new content is “*Although adopted by some scholars, the above methods are not suitable for this study because of the aforementioned shortcomings. Therefore, this paper employs the commonly used location quotient (LQ) to calculate the degree of industrial agglomeration. LQ is a very meaningful indicator to measure the spatial distribution of elements in a certain region and reflects the degree of specialization of a certain industrial sector. The greater the value of LQ, the greater the specialization rate. Munnich et al. (1998) adopted LQ as the measurement standard. Peters (2004) used LQ as the measurement standard. And he measured economic specialization for an industry in Missouri by calculating LQ for output, employment, compensation and foreign exports in 2000.* Jiang and Xu (2016) utilized location quotient (LQ) to measure the level of forestry industry agglomeration in Heilongjiang of China from the two perspectives of gross product and number of employees. *Zhang et al. (2016) employed LQ to measure the degree of industrial agglomeration taking industrial industries in different regions of China as research objects*”. The main measurement methods of industrial agglomeration are also important. Because we discussed the shortcomings of every methods after introducing these methods, for example, “the D-O index has very strict data requirements”. Finally, we decided not to delete the content on main methods. Regarding the part of “Linkages between industrial agglomeration and income inequality”, based on your suggestion, we have slightly adjusted the paragraphs to make the summaries clear. Additionally, for the part of “Income inequality and its determinants”, we have deleted the content on some variables not reflected in the empirical analysis. We also revised some variables’ names, such as “economic growth” to “*regional economic development level*” because both of them actually adopt the same index, to be consistent with the variables names in the empirical analysis of this paper. Based on your suggestion, the modified part of “Income inequality and its determinants” has been merged into the back of the part “Linkages between industrial agglomeration and income inequality”.

**Point 3:** Results and discussion: why was SPDM chosen as the empirical model of this paper? There are many models for testing nonlinear relationship. “Spatial effect of industrial agglomeration on income inequality” should be supplemented, especially, in the part of literature review. Lack of necessary testing analysis (e.g., Likelihood ratio test, Wald test, and etc.). The robustness test is unreasonable. Why is “service industry agglomeration” chosen to replace the independent variable? Does industry heterogeneity need further consideration? Why not choose other variables (e.g., manufacturing employment density) to substitute for the independent variable? You can also select some variables to substitute for the explained variable. The regression model of industrial agglomeration affecting income inequality will inevitably be troubled by the endogeneity problem to a certain extent. So, Endogeneity test may be warranted. Standardization also needs to be strengthened.

**Reply 3:** Thanks for your valuable comments. There are several reasons mentioned in the paper why this study chooses the spatial Durbin model. First, according to Moran’s *I*, the spatial autocorrelation of dependent and independent variables exists. Second, the industrial agglomeration not only affects income inequality in the province, but also income inequality of surrounding provinces. Income inequality between neighboring provinces also has spatial correlation and spatial spillover effects. Third, based on the suggestions of the reviewer, we have added testing analysis, such as Likelihood ratio test and Wald test, to verify why the spatial Durbin model is adopted. We present it in Table 4.

Table 4. Spatial econometric model testing.

| Model | *LM_Error* | *RLM_Error* | *LM_Lag* | *RLM_Lag* | *LR_SAR* | *LR_SEM* | *Wald_SAR* | *Wald_SEM* |
| --- | --- | --- | --- | --- | --- | --- | --- | --- |
| Spatial model | 51.348*** | 61.492*** | 0.155 | 10.3*** | 75.11*** | 68.18*** | 81.53*** | 73.22*** |

Note: *** *p* < 0.01, ** *p* < 0.05, * *p* < 0.1.

(**added manuscript**: *Additionally, a combination of LM_Error, RLM_Error (spatial error robustness test), LM_Lag and RLM_Lag (spatial lag robustness test) is adopted to further validate why this study uses the spatial Durbin model (SDM) rather than other common spatial models. The results are shown in Table 4. Based on the model without spatial effect (except LM_Lag), all the null hypotheses are rejected. Thus, SDM model is usually given priority because both the spatial autoregressive model (SAR) and the spatial errors model (SEM) can be accepted (Ellison et al., 2010). This paper also uses LR test and Wald test to prove whether SDM model can be degenerated into SAR model or SEM model. It is obvious that both LR value and Wald value reject the null hypothesis. Therefore, SDM model is suitable to study the effect of industrial agglomeration on income inequality from a spatial perspective*.)

There are few literatures on “Spatial effect of industrial agglomeration on income inequality”, thus, this is one of reasons that this paper investigates the impact of industrial agglomeration on income inequality from a spatial perspective. Regarding the robustness test, according to the reviewer’s suggestions, we have deleted “service industry agglomeration” and added manufacturing employment density to replace the independent variable. This paper uses maximum likelihood estimation (MLE) and the fixed effects to weaken the endogeneity problem. Furthermore, thanks for the reminding of the reviewer, we have added the one-period lagged manufacturing industrial agglomeration instead of the independent variable in robustness test to alleviate the potential endogeneity problem based on the research of Zhang et al. (2022). The revised robustness test is shown in Table 6. The highlighted parts are the added content.

Table 6. Robustness test of the spatial Durbin model.

| *inequ* | (III) | (Ⅴ) | (Ⅵ) | (Ⅶ) |
| --- | --- | --- | --- | --- |
| *magg* | 0.189*** |  |  | 0.163*** |
|  | (2.89) |  |  | (2.65) |
| ${magg}^{2}$ | -0.0812*** |  |  | -0.0764*** |
|  | (-2.91) |  |  | (-2.91) |
| ${magg}_{t-1}$ |  | 0.169** |  |  |
|  |  | (2.55) |  |  |
| ${magg}_{t-1}^{2}$ |  | -0.0752*** |  |  |
|  |  | (-2.67) |  |  |
| *lnmden* |  |  | 0.0271* |  |
|  |  |  | (1.92) |  |
| ${lnmden}^{2}$ |  |  | -0.0173*** |  |
|  |  |  | (-7.25) |  |
| *lnpergdp* | 1.211*** | 1.405*** | 0.933*** | 0.938*** |
|  | (8.07) | (8.40) | (6.24) | (6.26) |
| ${lnpergdp}^{2}$ | -0.0476*** | -0.0568*** | -0.0347*** | -0.0354*** |
|  | (-6.26) | (-6.75) | (-4.63) | (-4.64) |
| *unemp* | -0.0247*** | -0.0242*** | -0.0282*** | -0.0181*** |
|  | (-3.81) | (-3.61) | (-4.48) | (-2.91) |
| *lnGDR* | 0.0805*** | 0.0810*** | 0.0502** | 0.0836*** |
|  | (3.58) | (3.52) | (2.10) | (3.82) |
| *human* | -0.00930 | -0.00768 | -0.00953 | -0.00291 |
|  | (-1.57) | (-1.27) | (-1.63) | (-0.51) |
| *gov* | -0.170*** | -0.157*** | -0.195*** | -0.0234 |
|  | (-3.00) | (-2.77) | (-3.53) | (-0.46) |
| *lnsize* | -0.370*** | -0.350*** | -0.472*** | -0.438*** |
|  | (-6.85) | (-6.32) | (-8.74) | (-8.63) |
| *ρ* | 0.351*** | 0.365*** | 0.299*** | 0.308*** |
|  | (8.94) | (8.97) | (7.48) | (4.93) |
| *W*magg* | 0.172*** |  |  | 0.500*** |
|  | (4.58) |  |  | (7.47) |
| *W**${magg}_{t-1}$ |  | 0.168*** |  |  |
|  |  | (4.51) |  |  |
| *W*lnmden* |  |  | 0.0572*** |  |
|  |  |  | (3.64) |  |
| *W*lnpergdp* | -0.254*** | -0.261*** | -0.233*** | -0.206*** |
|  | (-11.60) | (-11.22) | (-10.20) | (-10.63) |
| *W*gov* | 0.222*** | 0.194*** | 0.140* |  |
|  | (3.01) | (2.59) | (1.91) |  |
| $R^{2}$ | 0.5868 | 0.5828 | 0.6308 | 0.6536 |

Note: t statistics in parentheses. *** *p* < 0.01, ** *p* < 0.05, * *p* < 0.1.

To make the description of variables clear and detailed, we have revised the table of variable description as follows. Some control variables’ maximums and minimums are very different, this study takes the logarithm of these variables. In Table 2, it can be seen that all variables’ standard deviations are small to meet standardization to a certain extend.

Table 2. Descriptive statistics.

| Variable | Mean | Std.Dev | Minimum | Maximum | Source |
| --- | --- | --- | --- | --- | --- |
| *inequ* | 1.013 | 0.439 | 0.511 | 2.750 | CSY |
| *magg* | 0.835 | 0.362 | 0.0938 | 1.828 | CSY |
| *lnpergdp* | 10.11 | 0.649 | 8.190 | 11.59 | CSY |
| *unemp* | 3.490 | 0.701 | 1.210 | 6.500 | CSY |
| *lnGDR* | 3.609 | 0.193 | 2.959 | 4.057 | CSY |
| *human* | 10.83 | 1.479 | 4.516 | 16.83 | CLSY |
| *gov* | 0.247 | 0.187 | 0.0768 | 1.379 | CSY |
| *lnsize* | 8.096 | 0.855 | 5.599 | 9.443 | CPESY |

Note: CSY, CLSY and CPESY represent China Statistical Yearbook, China Labour Statistical Yearbook and China Population and Employment Statistics Yearbook respectively.

**Point 4:** The paper is not innovative enough, and the research conclusions are not novel enough.

**Reply 4:** Thanks for your valuable comments. This paper mainly has the following innovations: Firstly, this paper presents an up-to-date portrait of the regional dimensions of income inequality across the country since the 21st century, which has been rarely depicted in the past. Secondly, most existing studies only focus on the linear impact of industrial agglomeration on income inequality, but the non-linear changes are not detailed enough. Industrial agglomeration affects income inequality through the agglomeration effect and crowding effect. It is easy to ignore the impact of the dynamic changes of the two effects on income inequality in the process of industrial agglomeration, which leads most studies to focus only on the linear relationship of the two variables. Therefore, this paper empirically investigates whether industrial agglomeration has a nonlinear relationship with income inequality. Thirdly, most previous studies have used traditional panel data analysis, ignoring the spatial correlation and spatial spillover effects of regional income inequality and industrial agglomeration. Thus, this paper uses global Moran’s *I* and local Moran’s *I* to test the spatial correlation of industrial agglomeration and income inequality respectively, then adopts the spatial Durbin model (SDM) to study the effect of industrial agglomeration on income inequality in China from 2003 to 2020. The research conclusions are based on employing the spatial models which are different from those of previous studies. Finally, based on the reviewer’s comments, we will make further efforts to optimize this paper.

We really appreciate for editors and reviewers’ warm work, and hope that the corrections will meet with approval. Once again, thank you very much for your comments and suggestions.

Sincerely,

Suhua Zhang

zhangsuhua1023@gmail.com
